# Hierarchical genetic interactions between FOXG1 and LHX2 regulate the formation of the cortical hem in the developing telencephalon

**DOI:** 10.1242/dev.154583

**Published:** 2018-01-01

**Authors:** Geeta Godbole, Ashwin S. Shetty, Achira Roy, Leora D'Souza, Bin Chen, Goichi Miyoshi, Gordon Fishell, Shubha Tole

**Affiliations:** 1 Department of Biological Sciences, Tata Institute of Fundamental Research, Mumbai 400,005, India; 2 Molecular, Cell and Developmental Biology, University of California Santa Cruz, CA 95064, USA; 3 Department of Neurobiology and the Stanley Center at the Broad, Harvard Medical School, Boston, MA 02115, USA

**Keywords:** LHX2, FOXG1, Telencephalon, Patterning, Hippocampus, Hem, Mouse

## Abstract

During forebrain development, a telencephalic organizer called the cortical hem is crucial for inducing hippocampal fate in adjacent cortical neuroepithelium. How the hem is restricted to its medial position is therefore a fundamental patterning issue. Here, we demonstrate that *Foxg1*-*Lhx2* interactions are crucial for the formation of the hem. Loss of either gene causes a region of the cortical neuroepithelium to transform into hem. We show that FOXG1 regulates *Lhx2* expression in the cortical primordium. In the absence of *Foxg1*, the presence of *Lhx2* is sufficient to suppress hem fate, and hippocampal markers appear selectively in *Lhx2*-expressing regions. FOXG1 also restricts the temporal window in which loss of *Lhx2* results in a transformation of cortical primordium into hem. Therefore, *Foxg1* and *Lhx2* form a genetic hierarchy in the spatiotemporal regulation of cortical hem specification and positioning, and together ensure the normal development of this hippocampal organizer.

## Abstract (precis)

**Summary:** The cortical hem, a telencephalic organizer that induces the hippocampus, is positionally restricted by the transcription factor FOXG1 acting directly and also by positively regulating LHX2.

## INTRODUCTION

The cortical primordium gives rise to the entire cerebral cortex, including both neocortex and hippocampus. The early patterning of the primordium is crucial for setting up a regulatory system that will control fundamental steps in neocortical and hippocampal development. Before cortical neurogenesis commences, a WNT- and BMP-expressing signalling centre called the cortical hem forms at the medial edge of the cortical primordium (Furuta et al., 1997; Grove et al., 1998). The hem is a secondary organizer in the embryo, necessary and sufficient for inducing the hippocampus in adjacent cortical neuroepithelium. Loss of the hem or of WNT3A, a hem-specific signal, results in loss of the hippocampus (Lee et al., 2000; Yoshida et al., 2006; Caronia-Brown et al., 2014), whereas ectopic hems are capable of inducing ectopic hippocampi (Mangale et al., 2008). This extrinsic signalling from the hem arises from the intrinsic identity of hem cells. Thus, the cell-intrinsic mechanisms that control the formation of the hem and restrict it to its medial location are of crucial importance because they determine the position of the hippocampus in the brain, and thus offer fundamental insights into the basic framework of early cortical patterning.

Based on studies of individual null mutant phenotypes, the transcription factors FOXG1 and LHX2 have been shown to regulate the formation of the hem. When either of these factors is constitutively lost, much of the dorsal telencephalic neuroepithelium transforms into hem instead of cortical primordium (Bulchand et al., 2001; Monuki et al., 2001; Vyas et al., 2003; Muzio and Mallamaci, 2005). There are, however, important differences in the two null mutant phenotypes. Although both display an expanded hem, the *Lhx2* mutant displays an expanded hem juxtaposed to an expanded anti-hem, with no cortical primordium in between them (Mangale et al., 2008). In contrast, loss of *Foxg1* spares only medial-dorsal fates, so the telencephalon contains an expanded hem and some hippocampal primordium, but no lateral cortical tissue or anti-hem (Vyas et al., 2003; Muzio and Mallamaci, 2005). Another important difference is that the specification of the ventral telencephalon appears normal in the *Lhx2* mutant, whereas this structure is entirely lost in the *Foxg1* mutant (Xuan et al., 1995; Huh et al., 1999; Martynoga et al., 2005).

Although FOXG1 and LHX2 have each been described as suppressors of hem fate, little attention has been paid to the interactions between them. Here, we uncovered new regulatory functions that position FOXG1 genetically upstream of LHX2 in the cascade responsible for the proper positioning of the hem. We also demonstrate, through analysis of *Foxg1* conditional loss of function, that LHX2 is the proximal suppressor of hem fate. Finally, we show that hippocampal specification always occurs in *Lhx2*-expressing tissue and is seen only adjacent to patches of hem. Our results provide insight into how a major component of the dorsal telencephalon, the hippocampus, is positioned based on its proximity to the hem.

## RESULTS AND DISCUSSION

We used mice carrying floxed alleles for *Foxg1* (Miyoshi and Fishell, 2012) and *Lhx2* (Mangale et al., 2008), and crossed them with a line expressing *CreERT2* constitutively from the *Rosa26* locus. *Foxg1* and *Lhx2* expression begins between E8.0 and E8.5 (Walther and Gruss, 1991; Hébert and McConnell, 2000; Tetreault et al., 2009), and by E12.5 both genes display robust expression in the dorsal telencephalon but are excluded from the hem (Bulchand et al., 2001; Muzio and Mallamaci, 2005) (Fig. S1). Hence, for experiments in which tamoxifen (Tam) was administered between E8.5 and E11.5, the age of examination was fixed at E12.5.

**Fig. 1.**
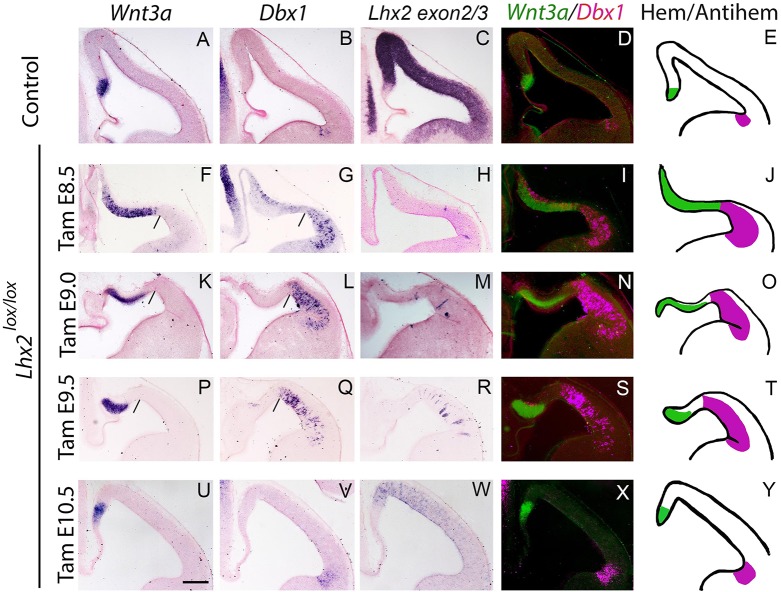
**Temporal analysis of hem and anti-hem suppression by LHX2.** (A-E) The expression of *Wnt3a* (hem), *Dbx1* (anti-hem) and *Lhx2 exon2/3* at E12.5 in control brains. (F-Y) Tamoxifen (Tam) was administered to *CreERT2; Lhx2^lox/lox^* embryos at E8.5 (F-J), E9.0 (K-O), E9.5 (P-T) or E10.5 (U-Y), and the embryos were harvested at E12.5. Both the anti-hem and hem expand up to a characteristic morphological ‘kink’ (black lines in F,G) when *Lhx2* is disrupted by tamoxifen administration at E8.5 (F-J). If administered at E9.0 or E9.5, the anti-hem continues to display a striking expansion up to the morphological kink (black lines in L,Q), but the hem appears progressively less expanded (K,P). *Lhx2* disruption at E10.5 does not cause either the anti-hem or hem to expand (U-Y). *Lhx2 exon 2/3* expression identifies the non-recombined cells in serial sections. False-colour overlays of the hem and anti-hem marker expression in each condition (D,I,N,S,X) and a schematized representation (E,J,OT,Y) are shown in each row. *Dbx1* expression above the hem in G and I is consistent with the presence of Cajal-Retzius cells from the expanded anti-hem, which line the surface of the entire dorsal telencephalon together with those derived from the expanded hem (Roy et al. 2014). Scale bar: 200 µm.

First, we examined the time window in which the hem and the anti-hem are sensitive to the loss of LHX2, as this transcription factor is known to suppress both fates. Administering tamoxifen to *CreER;Lhx2^lox/lox^* mice at E8.5 results in a phenotype that closely approximates that seen in *Lhx2*-null embryos in which the hem and anti-hem are both expanded and are seen juxtaposed to each other in the dorsal telencephalon, and no cortical primordium is detectable (Fig. 1F-J) (Mangale et al., 2008). Therefore, we chose E8.5 as the starting point for our analysis of loss of *Lhx2*, and E10.5 as the endpoint, as we and others had established in earlier work that loss of *Lhx2* after E10.5 does not result in expansion of the hem (Mangale et al., 2008; Chou et al., 2009). Disruption of *Lhx2* at E9.0 (Fig. 1K-O) reveals a disparity between the expansion of the hem and the anti-hem, and this disparity becomes more pronounced when tamoxifen is administered at E9.5 (Fig. 1P-T). Whereas the anti-hem continues to extend up to a characteristic morphological ‘kink’ in the mutant neuroepithelium (Fig. 1G,L,Q; black lines), the hem appears to be only minimally expanded, such that some medial cortical neuroepithelium is spared between the hem and the anti-hem. These data suggest that E9.5 represents a time point after which the medial neuroepithelium no longer requires LHX2 to prevent it from transforming into hem. By E10.5, the lateral neuroepithelium also appears to be insensitive to the loss of *Lhx2*. Administering tamoxifen at E10.5 does not appear to alter the normal extent of either the hem or the anti-hem (Fig. 1U-Y).

In summary, we demonstrate that the critical period for LHX2-mediated suppression of the hem ends earlier than that for suppression of the anti-hem (summarized in Table S1). This is surprising as the neurogenetic gradient in the dorsal telencephalon progresses in a lateromedial direction; therefore, one would expect lateral fates to be established prior to medial fates. One possible explanation for this is that the anti-hem, but not the hem, is subject to patterning interactions between PAX6 and GSX2 (Torresson et al., 2000; Yun et al., 2001), and these modulatory effects may affect the timing of its regulation by LHX2 through mechanisms that are not yet clear.

The loss of *Foxg1* also causes an expansion of the hem, but this is the only similarity it shares with the *Lhx2* mutant phenotype. Upon loss of *Foxg1*, the entire dorsal telencephalon is respecified as medial pallium, so that only hem, hippocampus, and Cajal-Retzius cells that are derived from the hem are present (Muzio and Mallamaci, 2005). There is also a loss of the entire ventral telencephalon in the absence of FOXG1, owing to a profound deficit of cell proliferation in this tissue after E9.5, accompanied by a lack of ventral telencephalic SHH. Together, these deficits account for the severely abnormal morphology of the E12.5 brain (Xuan et al., 1995; Huh et al., 1999; Martynoga et al., 2005). Nonetheless, the presence of an expanded hem motivated the hypothesis that *Lhx2* and *Foxg1* may interact to restrict this domain. First, we ascertained that administration of tamoxifen at E8.5 to *CreER; Foxg1^lox/lox^* animals recapitulates the well-established *Foxg1*-null phenotype of loss of ventral and lateral telencephalic tissue. Indeed, we find that *Wnt8b* expression, which is normally restricted to the hem and hippocampal primordium, encompasses the entire telencephalic neuroepithelium in both *Foxg1*-null and *Foxg1 ^lox/lox^* embryos (Fig. S2).

**Fig. 2.**
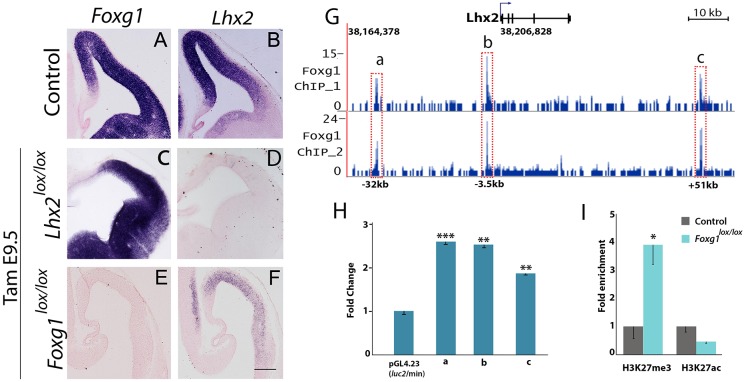
**FOXG1 regulates the expression of *Lhx2* in the dorsal telencephalic neuroepithelium.** (A-F) Tamoxifen (Tam) was administered at E9.5 to control, *CreERT2;Foxg1^lox/lox^* and *CreERT2;Lhx2^lox/lox^* embryos, which were examined at E12.5. (A,B) *Foxg1* and *Lhx2* are normally highly expressed in the dorsal telencephalon in control embryos. (C,D) The expression of *Foxg1* is unaltered in the absence of *Lhx2*, and the extent of *Lhx2* recombination is revealed by a probe against the floxed exon. (E,F) *Foxg1* conditional mutants display no detectable expression of *Foxg1*, and a profound reduction in *Lhx2* expression. (G) FOXG1 ChIP-seq revealed three MACS peaks (model-based analysis of ChIP-seq) denoted by a, b and c that are associated with the *Lhx2* gene locus. (H) A luciferase assay was performed using constructs that contained either regions corresponding to the a, b or c peaks, upstream of a vector containing a minimal promoter and a luciferase reporter. Each of the constructs produces a significant upregulation of the reporter in response to the addition of FOXG1 (*n*=4, **P<0.01, ***P<0.001; Student's *t*-test). (I) Histone ChIP was performed on chromatin isolated from E12.5 *CreERT2; Foxg1^lox/lox^* tissue (Tamoxifen at E10.5), using anti-PanH3, anti- H3K27Ac and anti-H3K27me3 antibodies. A significant increase in the repressive H3K27me3 mark and an apparent decrease in the activatory H3K27Ac mark were seen at the LHX2 TSS region (*n*=3, *P<0.05, Student's *t*-test). PanH3 levels were used to normalize the data. Scale bar: 200 µm.

To test whether FOXG1 and LHX2 regulate expression of each other in the cortical primordium, we selected E9.5 for tamoxifen administration, as loss of *Lhx2* at this stage spares the medial cortical primordium and does not cause it to become converted to hem (Fig. 1). When *Lhx2* is lost from E9.5, *Foxg1* expression is similar to that seen in controls. Therefore, loss of *Lhx2* does not appear to affect *Foxg1* expression in the cortical primordium (Fig. 2A-D; additional embryos in Fig. S3). This result is qualitatively distinct from our previous study (Mangale et al., 2008), in which *Lhx2^null^* patches among *Lhx2^+/+^* tissue formed ectopic hems that did not express *Foxg1*, in the medial telencephalon. In the ectopic hems of Mangale et al. (2008), downregulation of *Foxg1* may occur because it depends critically on LHX2 to maintain its expression in medial tissue prior to E8.5. After E9.5, other factors may regulate *Foxg1* and compensate for loss of LHX2, resulting in no detectable change in *Foxg1* expression.

In contrast, loss of *Foxg1* results in a marked reduction of *Lhx2* expression (Fig. 2E,F; additional embryos in Fig. S4). These findings indicate FOXG1 may regulate *Lhx2* directly or indirectly. We performed ChIP-­seq using anti-­FOXG1 antibody on chromatin isolated from E14.5 cortical tissue and identified three sites of FOXG1 occupancy in the region of the *Lhx2* locus (Fig. 2G). Two of these are at some distance away from the *Lhx2* transcription start site (TSS; Refseq Accession number: NM_010710), at −32 kb and +51 kb (Fig. 2G, peaks ‘a’ and ‘c’, respectively). One site is closer to the TSS, at −3.5 kb (Fig. 2G, peak ‘b’). To test the hypothesis that FOXG1 regulates LHX2 directly, we performed a luciferase assay using fragments corresponding to each of the a, b and c peaks individually, upstream of a minimal promoter that drives the luciferase reporter construct. Each fragment gave a significant induction of the luciferase reporter in the presence of *Foxg1* (Fig. 2H). These results indicate that the occupancy regions we identified on the *Lhx2* locus are indeed sites via which FOXG1 is able to positively regulate its target. Although the ChIP-seq was performed in E14.5 tissue, the accompanying luciferase assay also demonstrates the ability of Foxg1 to regulate *Lhx2* in the context of a heterologous system. Taken together, these finding indicate that the Foxg1-binding sites we identified may also be relevant to the regulation of Lhx2 at earlier stages such as E9.5.

We further tested for changes in histone modification marks at the *Lhx2* TSS upon loss of *Foxg1*. Chromatin was isolated from control and *Foxg1^lox/lox^* dorsal telencephalic tissue harvested at E12.5. In the absence of *Foxg1*, the *Lhx2* TSS displays a fourfold increase in levels of the repressive H3K27me3 mark and an apparent reduction in levels of the activatory H3K27 acetyl mark compared with controls (Fig. 2I). Together, these data indicate that FOXG1 regulates *Lhx2* directly. This is the first evidence of a direct upstream regulator for *Lhx2* in the mammalian brain.

**Fig. 3.**
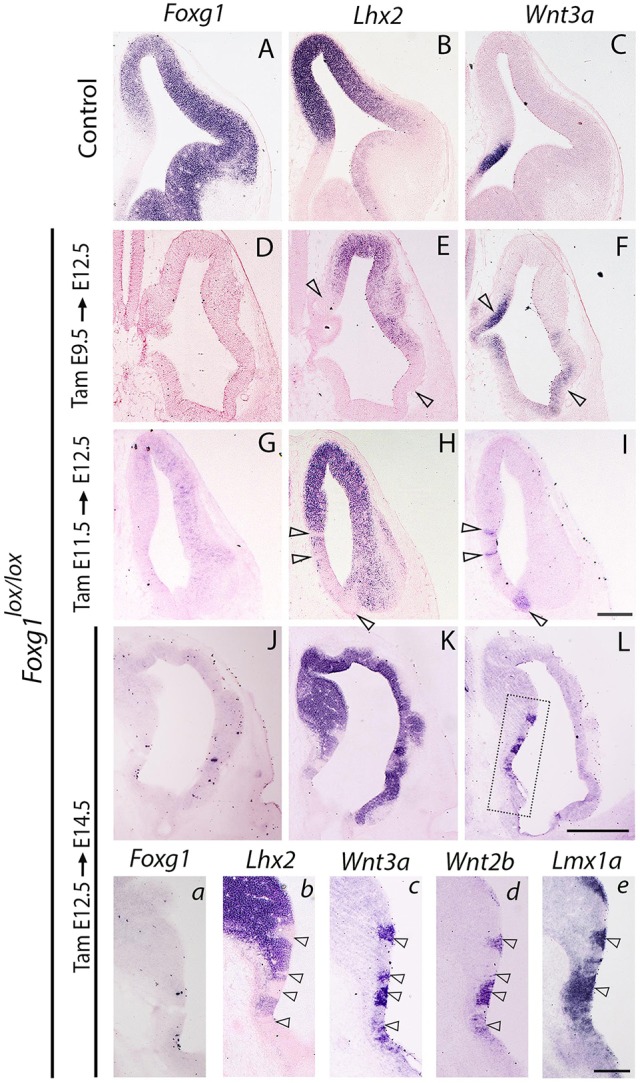
***Lhx2* restricts the hem domain in a *Foxg1 mutant* background.** (A-C) Expression of *Foxg1*, *Lhx2* and *Wnt3a* in control brains. (D-I) Tamoxifen (Tam) was administered at E9.5 (D-F) or E11.5 (G-I) to control and *CreERT2; Foxg1^lox/lox^* embryos, and brains were harvested at E12.5. Regions devoid of *Lhx2* expression correspond to ectopic hem patches marked by *Wnt3a* expression (open arrowheads, E,F,H,I). (J-L) Tamoxifen was administered to *CreERT2; Foxg1^lox/lox^* at E12.5 and the embryos were harvested at E14.5. (a-e) Magnified views of serial sections corresponding to the area within the box in L. When *Foxg1* is removed at E12.5 (J,a), *Lhx2* expression is lost in patches (K; b, arrowheads). These patches express the hem markers *Wnt3a* (L; c, arrowheads), *Wnt2b* (d, arrowheads) and *Lmx1a* (e, arrowheads). Sections a-e are in a continuous series, so a comparison between b (*Lhx2*) and c (*Wnt3a*) shows the alignment of hem patches in immediately adjacent sections. Scale bars: 200 μm for A-I; 400 µm for J-L; 100 µm for a-e.

We next examined whether the apparent regulation of *Lhx2* by FOXG1 is dependent on the stage of *Foxg1* removal. Upon administration of tamoxifen at E9.5 to *Foxg1^lox/lox^* animals, which results in near-­complete recombination of *Foxg1*, *Lhx2* expression levels appear much weaker than in control brains, and some regions display no detectable *Lhx2* expression (Fig. 3D,E; additional embryos in Fig. S5). It is noteworthy that only *Lhx2*-negative regions display hem markers, whereas *Foxg1*-negative regions do not do so as long as there is a detectable level of *Lhx2* expression. Surprisingly, this is also seen when *Foxg1* is disrupted at E11.5. Although the overall *Lhx2* levels seem less affected (Fig. 3G-I; additional embryos in Fig. S6), loss of *Foxg1* from E11.5 nevertheless causes some *Lhx2*-negative patches to form. Therefore, the ability of FOXG1 to regulate *Lhx2* levels is strongest when *Foxg1* is removed from E9.5. Intriguingly, regardless of the stage of *Foxg1* removal, discrete *Lhx2*-negative domains are seen in the neuroepithelium, and these display hem markers. The presence of *Lhx2*-negative patches of tissue, regardless of when *Foxg1* is removed, indicates that FOXG1 regulates *Lhx2* expression, and its loss reliably causes downregulation of *Lhx2* in some populations.

It appears that, in the absence of FOXG1, the critical period in which LHX2 suppresses hem fate is expanded beyond E9.5, suggesting that FOXG1 in itself is crucial for suppressing cortical plasticity (Hanashima et al., 2004). We tested this hypothesis further, and administered tamoxifen to *Foxg1^lox/lox^* animals at E12.5, well after the hem has formed, and examined the embryos at E14.5. Surprisingly, we discovered ectopic patches of hem (Fig. 3J-L). These patches appeared only at very rostral levels of sectioning, and once again correlated with patches in which *Lhx2*-negative cells had accumulated. We ascertained that these patches were transformed to hem fate by examining three hem markers, *Wnt3a*, *Wnt2b* and *Lmx1a*, in serial sections. All three markers identify similar territories as ectopic hem (Fig. 3C-E and Fig. S7). Thus, FOXG1 appears to limit the critical period during which LHX2 suppresses hem fate; upon loss of *Foxg1*, this time window is expanded to as late as E12.5. These data are summarized in Table S1. It is important to note that although CreER is known mediate recombination within 6 h of tamoxifen administration, the actual loss of functional FOXG1 or LHX2 protein would depend on the half-life of both the mRNA and the stability of the protein that has already accumulated. For multifunctional factors such as these, we expect that the turnover of mRNA and protein would be comparable with the cell cycle duration of ∼8 h at E10.5 (Quinn et al., 2007) so as to ensure tight regulation of downstream targets during subsequent cell divisions. Consistent with this interpretation, administration of tamoxifen to *Foxg1^lox/lox^* at E11.5 results in *Lhx2*-negative patches by E12.5 that have transformed into ectopic hems (Fig. 3G,H). This indicates that 24 h is sufficient for: (1) CRE-mediated recombination of the *Foxg1* locus; (2) the resulting drop in *Foxg1* mRNA and protein levels to occur; (3) a consequential reduction in *Lhx2* mRNA transcription to occur that results in LHX2 protein reaching sub-threshold levels; and (4) the transcription of hem markers to finally reach levels that are detectable by *in situ* hybridization.

Regardless of the stage or extent of *Foxg1* disruption, one striking result is that hem only forms where *Lhx2* is completely undetectable. Regions of even very weak *Lhx2* expression are complementary and completely non-overlapping with regions where hem is formed. This suggests that LHX2 is a proximal and highly effective suppressor of hem fate. Furthermore, hem does not form where *Foxg1* is undetectable but *Lhx2* is present, indicating that LHX2 is downstream of FOXG1 in restricting the hem to its location. However, it is not completely clear why only some cells entirely lose *Lhx2* expression after *Foxg1* deletion. It is possible that there is a threshold effect, such that LHX2 levels falling below a certain level triggers hem fate, which could then cause suppression of all remaining *Lhx2* expression in those cells.

**Fig. 4.**
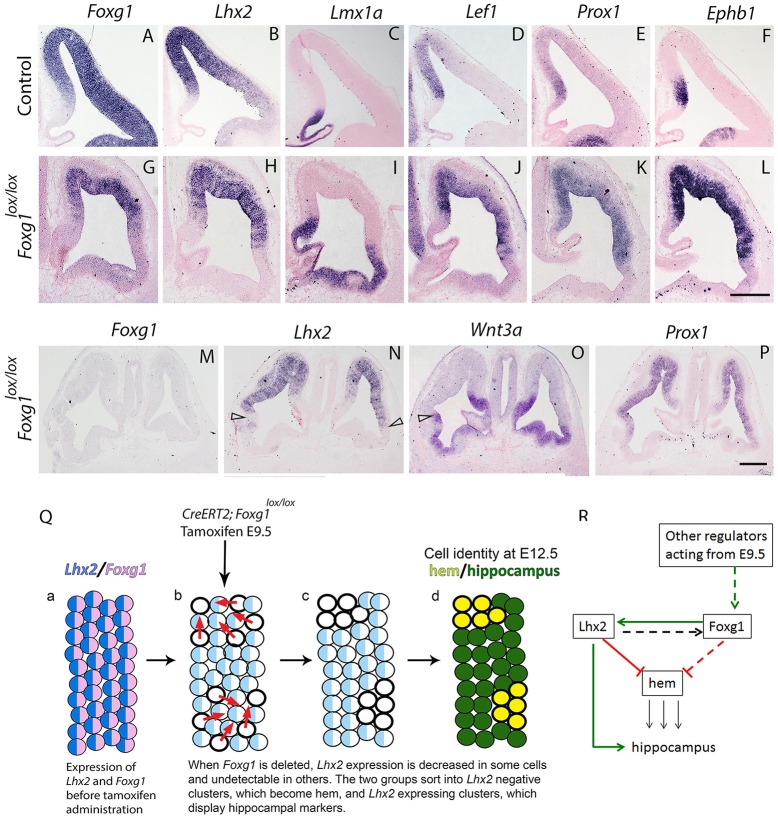
**Ectopic hippocampal markers appear adjacent to the ectopic hem.** (A-F) Control brains at E12.5 display hippocampal primordium markers *Lef1*, *Prox1* and *Ephb1* in neuroepithelium that also expresses *Foxg1* and *Lhx2.* (G-P) Tamoxifen was administered at E9.5 to *CreERT2; Foxg1^lox/lox^* embryos, and brains were harvested at E12.5. (G) *Foxg1* expression is present in dorsal regions that escaped recombination, but not in ventral regions. (H) *Lhx2* displays a similar pattern to FOXG1. (I) Ectopic hem identified by *Lmx1a* expression is seen in regions devoid of *Foxg1* and *Lhx2*. (J-L) The hippocampal primordium markers *Lef1*, *Prox1* and *Ephb1* are seen in tissue that excludes the ectopic hem and expresses *Foxg1* and *Lhx2*. (M-P) Another brain with complete recombination of *Foxg1* (M) and reduced expression of *Lhx2* (N). In the complete absence of *Foxg1*, ectopic hem is seen only regions lacking *Lhx2* expression (arrowheads, N,O) and hippocampal marker *Prox1* appears only in regions displaying *Lhx2* expression. Scale bars: 200 µm. (Q) Summary schematic. (a) Cortical neuroepithelial cells express *Foxg1* (pink) and *Lhx2* (blue). (b-d) Removal of *Foxg1* by tamoxifen administration at E9.5 causes a decrease in *Lhx2* expression in some cells (light blue) and complete loss of *Lhx2* in some cells (open circles). *Lhx2*-negative cells cluster together (red arrows, b) and transform into hem (yellow, d), whereas *Lhx2-*expressing cells also cluster together, and express hippocampal markers when they lie adjacent to the hem patches (green, d). This provides a molecular basis for the *Foxg1* mutant phenotype in which only hem and hippocampal fates are seen. (R) Model of interactions. Solid red and green lines indicate interactions based on data in this study. Broken red and green lines represent regulatory effects hypothesized based on data in this study, and the dashed black line represents a predicted regulation of *Foxg1* by LHX2 in the medial telencephalon, based on data from Mangale et al. (2008). Black lines indicate inductive effects.

In earlier work, we demonstrated that *Lhx2*-null cells ‘prefer’ to aggregate with each other and exclude wild-type cells, and vice versa (Mangale et al., 2008). To our knowledge, this phenomenon appears to be unique to loss of LHX2 and limited to the embryonic dorsal telencephalon. In contrast, the ventral telencephalon in *Lhx2^lox/lox^* brains appears completely floxed when the dorsal telencephalon displays *Lhx2*^+^ and *Lhx2*^−^ patches (three embryos in Fig. S3). In our current experiments, it appears that cells that have downregulated *Lhx2* as a result of loss of *Foxg1* behave similarly, and form clusters separated by those that express *Lhx2*. As LHX2 suppresses hem fate, clusters of cells that have downregulated *Lhx2* transform into hem. The apparent homotypic aggregation of *Lhx2*^−^ cells could be a mechanism that defines and maintains a key boundary between the hem and the hippocampal primordium: the organizer and the responsive tissue, respectively (Mangale et al., 2008). Indeed, when we examined whether hippocampal markers arise adjacent to the hem patches seen in *Foxg1* single conditional mutants, *Prox1*, *Lef1* and *Ephb1* all appeared in ectopic locations adjacent to the ectopic hem patches (Fig. 4). Hippocampal markers appear only in tissue that expresses *Lhx2*, and it does not seem to matter whether *Foxg1* is present or not (Fig. 4 shows two embryos displaying different extents of loss of *Foxg1* due to some variability in recombination of the *Foxg1^lox/lox^* allele). Therefore, LHX2, but not FOXG1, is crucial for hippocampal fate specification. It is surprising that weak *Lhx2* expression supports hippocampal specification given the normally high levels of LHX2 expression seen in the hippocampal primordium (Bulchand et al., 2001) (Fig. S1). Together, the results suggest a single threshold for LHX2 levels, below which hem fate is triggered, and above which hippocampal specification is possible.

Perhaps the most unexpected result in our study is that removing *Lhx2* alone prior to E9.5 produces ectopic hem (Fig. 1) (Mangale et al., 2008), whereas removing *Lhx2* after E9.5 does not do so unless *Foxg1* is also removed (Figs 3 and 4). As schematized in Fig. 4R, we propose possible interactions that could explain this intriguing finding. (1) We suggest that LHX2 is required for *Foxg1* expression in the medial telencephalon prior to E9.5, but it is redundant, i.e. substituted by other regulators of *Foxg1*, from E9.5 onwards. (2) It is also possible that, in addition to an indirect role via LHX2, FOXG1 is capable of suppressing hem directly. If this were true, then loss of both factors would be necessary to induce ectopic hem. The pre- and post-E9.5 scenarios would be as follows: loss of *Lhx2* prior to E9.5 apparently causes loss of *Foxg1* in medial tissue, and therefore produces ectopic hem medially, consistent with our previous findings (Mangale et al., 2008). In contrast, after E9.5, loss of *Lhx2* is no longer sufficient to cause loss of *Foxg1* (Fig. 2), therefore ectopic hems cannot form, as we demonstrate in Fig. 1. However, removal of *Foxg1* causes loss of *Lhx2* in some cells, and these double-negative cells then aggregate to form ectopic hem, which is consistent with our findings in Fig. 3.

In summary, our work suggests both hierarchy and synergy in the interactions between the fundamental regulators of cortical development, FOXG1 and LHX2, that is not obvious from examination of individual loss-of-function phenotypes. We also demonstrate that FOXG1 is a direct upstream regulator of *Lhx2*. Together with studies in the zebrafish, where LHX2 mediates the activity of SIX3 in regulating forebrain size (Ando et al., 2005), it appears that LHX2 functions at a key node in the network that controls the earliest developmental decisions required for telencephalic patterning. Our findings suggest avenues for future studies that could focus on this fascinating transcription factor biology at the earliest stages of telencephalic patterning that sets the stage for subsequent stages of cerebral cortical development.

## MATERIALS AND METHODS

### Mice

All animal protocols were approved by the Institutional Animal Ethics Committee (Tata Institute of Fundamental Research, Mumbai, India) according to regulations devised by the Committee for the Purpose of Control and Supervision of Experiments on Animals (CPCSEA), India. The tamoxifen-inducible *CreERT2* line [strain name: B6; 129-Gt(ROSA)26Sortm1(*Cre/ERT*)Nat/J; stock number: 004847] was obtained from the Jackson Laboratory. The *Lhx2 ^lox/lox^* line used in this study was obtained from Edwin Monuki (University of California, Irvine, CA, USA) (Mangale et al., 2008). The *Foxg1 ^lox/lox^* line has been described previously (Miyoshi and Fishell, 2012). Three or more embryos of each genotype were examined. For each embryo, the extent of recombination was examined in one series of sections. The stage of tamoxifen administration varied from E8.5 to E12.5; however, all embryos were examined at E12.5, except the E12.5 tamoxifen timepoint, which was analysed at E14.5.

Noon of the day of vaginal plug was designated as embryonic day 0.5 (E0.5). Tamoxifen (Sigma) was administered to the pregnant dams at different time points, as mentioned in the text, and embryos were harvested at E12.5. Control embryos were littermates with one wild-type copy of the relevant gene. For *Lhx2 ^lox/lox^* mice, the tamoxifen dose administered was 75 µg/gm body weight. *Foxg1 ^lox/lox^* mice were found to be sensitive to tamoxifen toxicity, so lower doses were tested, and the tamoxifen dose used in the experiments was 28 µg/g body weight of the animal. We ascertained that extensive recombination was seen 6 h post tamoxifen administration using an Ai9 reporter (Fig. S8).

### Sample preparation and *In situ* hybridization

Freshly harvested brains were fixed in 4% paraformaldehyde (PFA) (Sigma) overnight and equilibrated in 15% sucrose followed by 30% sucrose (SRL Chem). The brains were sectioned at 16 µm sections using a freezing microtome and mounted on Superfrost Plus slides (Electron Microscopy Sciences). Sections were post-fixed in 4% PFA, washed in phosphate-buffered Saline (PBS) and treated with proteinase K (Sigma) (1 µg/ml) at 37°C for 10 min. One more round of post-fixing and PBS washes was performed, and the sections were incubated in hybridization buffer (5×SSC, 50% formamide, 1% SDS) containing different antisense RNA probes at 70°C overnight. Probes were prepared using a kit according to the manufacturer's instructions (Roche). The next day, after washes with solution X (2×SSC, 50% formamide, 1% SDS) at 70°C, followed by stringent washes with 2×SSC and 0.2×SSC at room temperature, the sections were washed with TBST [25 mM Tris-HCl (pH 7.5) and 150 mM NaCl, 0.1% KCl, 0.5% Tween-20]. The slides were then incubated with alkaline phosphatase-conjugated anti-digoxigenin Fab fragments (1:5000, Roche) for 16 h at 4°C. The slides were then washed four times with TBST and then with developing buffer NTMT [100 mM NaCl, 100 mM Tris (pH 9.5), 50 mM MgCl, 1% Tween-20]. The colour reaction was performed using Nitro-blue tetrazolium chloride and 5-bromo-4-chloro-3-indolyl-phosphate (NBT-BCIP, Roche) according to the manufacturer's instructions. The incubation was performed for 10-40 h and terminated when the colour reaction had developed satisfactorily, as assessed by the intensity of signal and low background. The reaction was stopped using Tris-EDTA [10 mM Tris-HCl (pH 7.5), 10 mM EDTA (pH 8.0)] and fixed 3.7% formaldehyde [diluted in PBS from a 37% stock (Sigma)] for 1 h at room temperature. Finally, the slides were washed in PBS, dried and mounted in DPX mountant (S.D Fine Chem).

To identify unrecombined cells, RNA probes against *Lhx2* exon2/3 were made by PCR followed by *in vitro* transcription (Roche) according to the manufacturer's instructions. All *in situ* hybridization experiments were performed on at least three brains of each genotype, for every time point of tamoxifen administration. For each embryo, the extent of recombination was examined by *in situ* hybridization for the recombined exon in one series of sections.

### ChIP-seq

Chromatin immunoprecipitation (ChIP) followed by sequencing was performed on E14.5 cerebral cortices from wild-type embryos using FOXG1 antibody (Santa Cruz Biotechnology) as described previously (Kumamoto et al., 2013). Two independent ChIP experiments were sequenced on an Illumina Hiseq 2000 platform. FOXG1 binding peaks were identified using MACS (Model-based Analysis of ChIP-Seq) and the binding motif was identified using MEME-ChIP analysis (Dreme: discriminative regular expression motif elicitation) (Kumamoto et al., 2013). The *Lhx2* transcriptional start site (TSS) was obtained using Refseq (accession number NM_010710).

### Chromatin immunoprecipitation and qPCR

Tamoxifen was administered at E9.5 and cortices were dissected from E12.5 *CreERT2; Foxg1 ^lox/lox^* and littermate control embryos. Cortices from two embryos were pooled for each sample. Three independent experiments were performed (biological replicates). The tissue was fixed for 5 min with 1% formaldehyde and quenched with 125 mM glycine. Cells were lysed and the chromatin was sheared into ≥200 bp fragments using a Covaris S220 sonicator (15 cycles: 60 s on/30 s off) in SDS lysis buffer. Immunoprecipitation was performed with Protein A and G Dynabeads (Invitrogen) mixed in equal proportions and using antibodies against H3K27acetyl (Diagenode), H3K27 trimethyl (Diagenode), rabbit IgG (Sigma) and panH3 control (Abcam) in the ratio 1 μg antibody for every 5 μg of chromatin. The immunoprecipitated DNA was purified using phenol-chloroform-isoamyl alcohol (Ambion). Equal amounts of input and immunoprecipitated DNA were used for quantitative PCR (qPCR) using SYBR Green (Light Cycler 480, Roche).

The primer sequences used were: LHX2 F, 5′ GATGTAGCTGCCCCCACGCC 3′; and LHX2 R, 5′TGTGGAACAGCATCGCGGC 3′. These primers span the region from −206 to +13 bps at the *Lhx2* TSS. Each sample was run in duplicates for qPCR, and the average of these technical replicate Ct readings was used for further calculation. Wild-type control and *Foxg1^lox/lox^* Ct values were normalized to their respective panH3 Ct values. The fold enrichment was calculated as a ratio of 2^-ΔCt^. Data from control and *Foxg1^lox/lox^* samples from the three independent experiments was compared using Student's unpaired *t*-test to determine statistical significance. Statistical analyses were performed using Microsoft Excel and values are expressed as mean±s.e.m.

### Cloning the FOXG1 occupancy sites on Lhx2 locus in the pGL4.23[*luc2*/minP] vector

Primers were designed for amplifying the regions corresponding to the three peaks seen in the ChIP-seq occupancy profile, marked a, b and c in Fig. 2. Genomic DNA was used as the template. The primer sequences are as mentioned in Table 1. For cloning regions ‘a’ and ‘c’, *Kpn*I and *Xho*I were used; for cloning region ‘b’, *Sac*I and *Xho*I were used to digest pGL4.23[*luc2*/minP] (Promega, E8411) at the MCS.

**Table 1.**
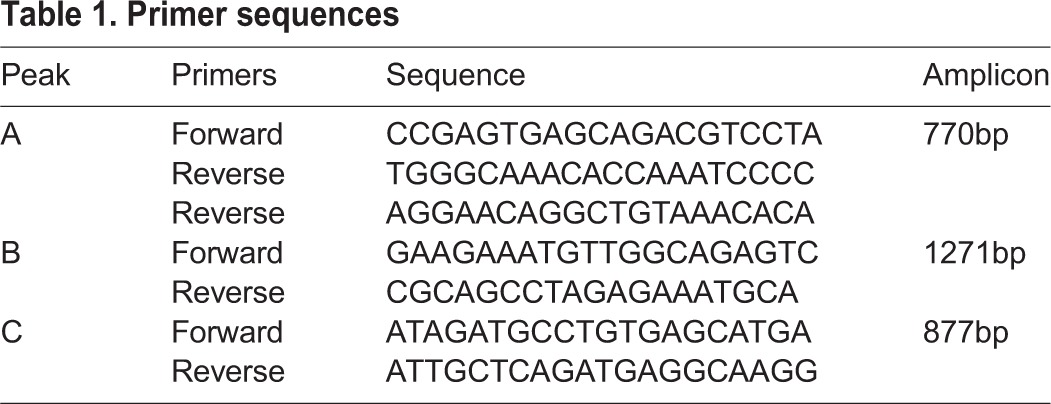
**Primer sequences**

### Luciferase assay

U-87 MG cells (a kind gift from Neelam Shirsat, The Advanced Centre for Treatment, Research and Education in Cancer, Navi Mumbai, India) were cultured in DMEM supplemented with 10% (vol/vol) FBS, glutamax, penicillin (100 U/ml) and streptomycin (100 μg/ml). For transfections, 5×10^5^ cells per well were seeded into 24-well plates. After an overnight incubation, the medium was removed and replaced with DMEM supplemented with 10% (vol/vol) FBS, glutamax without antibiotics for 2 h. After 2 h, the DMEM medium was replaced with transfection medium. For transfection, the pGL4.23[*luc2*/minP] vector with individually cloned FOXG1 occupancy regions (a, b and c) was added with either *GFP* (control) or *Foxg1*-*GFP* (*pCAGGS-Foxg1*) (500 ng/well for each construct) together with Renilla luciferase vector (100 ng/well) as a normalization control. Transfection was performed using 0.5 µl PLUS reagent and 2 μl of lipofectamine-LTX (Invitrogen) in 400 µl of optimem medium per well and incubated for 6 h. After 6 h, the transfection medium was replaced with fresh complete medium with antibiotics. Luciferase activity was measured using a commercial dual-Glo luciferase assay system (Promega, E2920) 48 h after transfection using Tecan Infinite Lumi Plate reader. The firefly luciferase readouts were normalized to their respective Renilla luciferase readouts obtained from the same cell lysate. The fold change in response to FOXG1 activity was calculated with respect to EGFP controls. All the values are expressed as the mean±s.e.m. of four biological replicates.

## Supplementary Material

Supplementary information

Supplementary information
