# Supplementary material for: Hierarchical genetic interactions between FOXG1 and LHX2 regulate the formation of the cortical hem in the developing telencephalon
Source: Development. 2018 Jan 1;145(1):dev154583. doi: 10.1242/dev.154583 (PMC5825872; doi:10.1242/dev.154583)
Supplement: Supplementary information [file develop-145-154583-s1.pdf]

Supplementary Figures

Figure S1

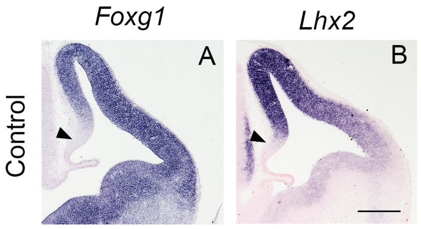

Figure S1: **Expression of *Foxg1* and *Lhx2* at E12.5 in control embryos**. (A,B) shows the expression pattern of *Foxg1* and *Lhx2*, excludes the hem (arrowheads) in control brain sections at E12.5. (A) *Foxg1* is expressed in the medial and lateral dorsal telencephalic neuroepithelium. (B) *Lhx2* is expressed in a medial (high), lateral (low) gradient. Scale bar is 200µm.

Development • Supplementary information

Figure S2

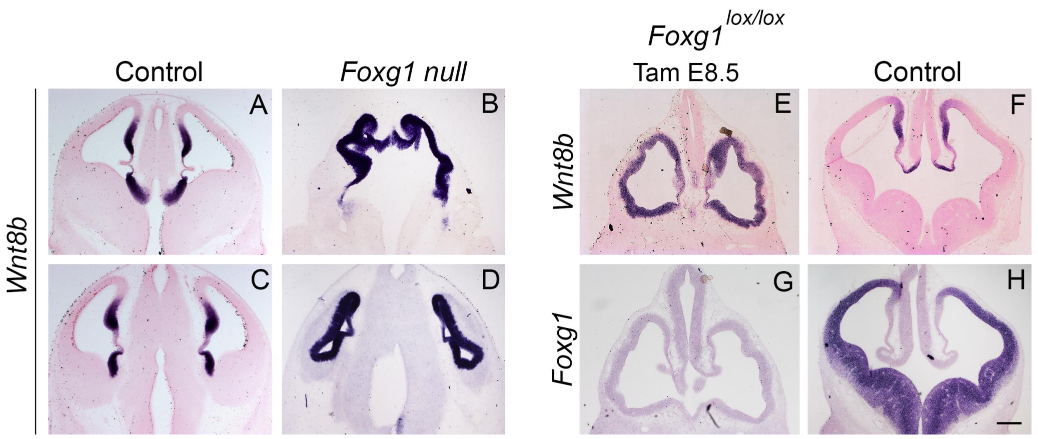

Figure S2: Medial fates occupy the entire telencephalon in the absence of *Foxg1*

(A-D);*Wnt8b* expression at E12.5 in control brains is limited to the medial telencephalon (A,C) whereas in *Foxg1* null mutant brains (B,D) it encompasses the entire telencephalic neuropeithelium. Two levels of sectioning are shown corresponding to (A,B) and (C,D) respectively. (E-H); When tamoxifen (Tam) is administered at E8.5 to *CreERT2; Foxg1* $^{lox/lox}$ mice, a similar pan-telencephalic expression of *Wnt8b* is seen (E). An absence of *Foxg1* expression confirms near-complete recombination (G). The expression of *Wnt8b* and *Foxg1* in control littermates is shown in (F,H) respectively. Scale bar is 200µm.

Development • Supplementary information

Figure S3

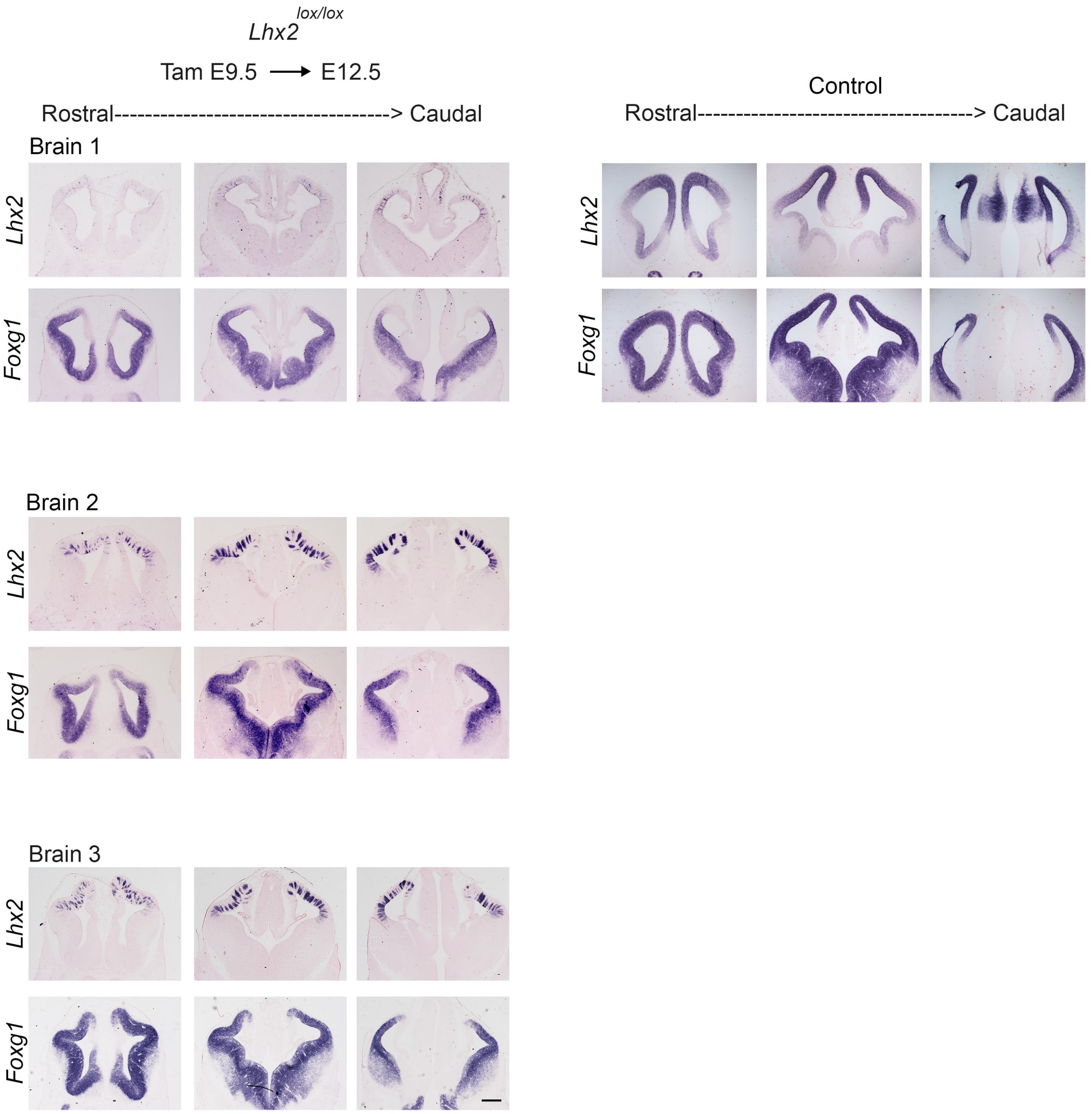

Figure S3: *Foxg1* expression is unaltered when *Lhx2* is removed from E9.5 (Additional embryos corresponding to Figure 2 C,D)

Tamoxifen (Tam) was administered at E9.5 to control and *CreERT2; Lhx2*$^{lox/lox}$ embryos and brains were harvested at E12.5. Sections from three embryos are presented showing the rostro-caudal expression profiles of *Foxg1* in *Lhx2* conditional mutants. Patches of *Lhx2* expression corresponding to regions that escaped recombination are detected using a probe specific to the floxed exon. The control panel on the right shows the normal rostro-caudal expression profiles of *Foxg1* and *Lhx2*. Scale bar is 200µm.

Figure S4

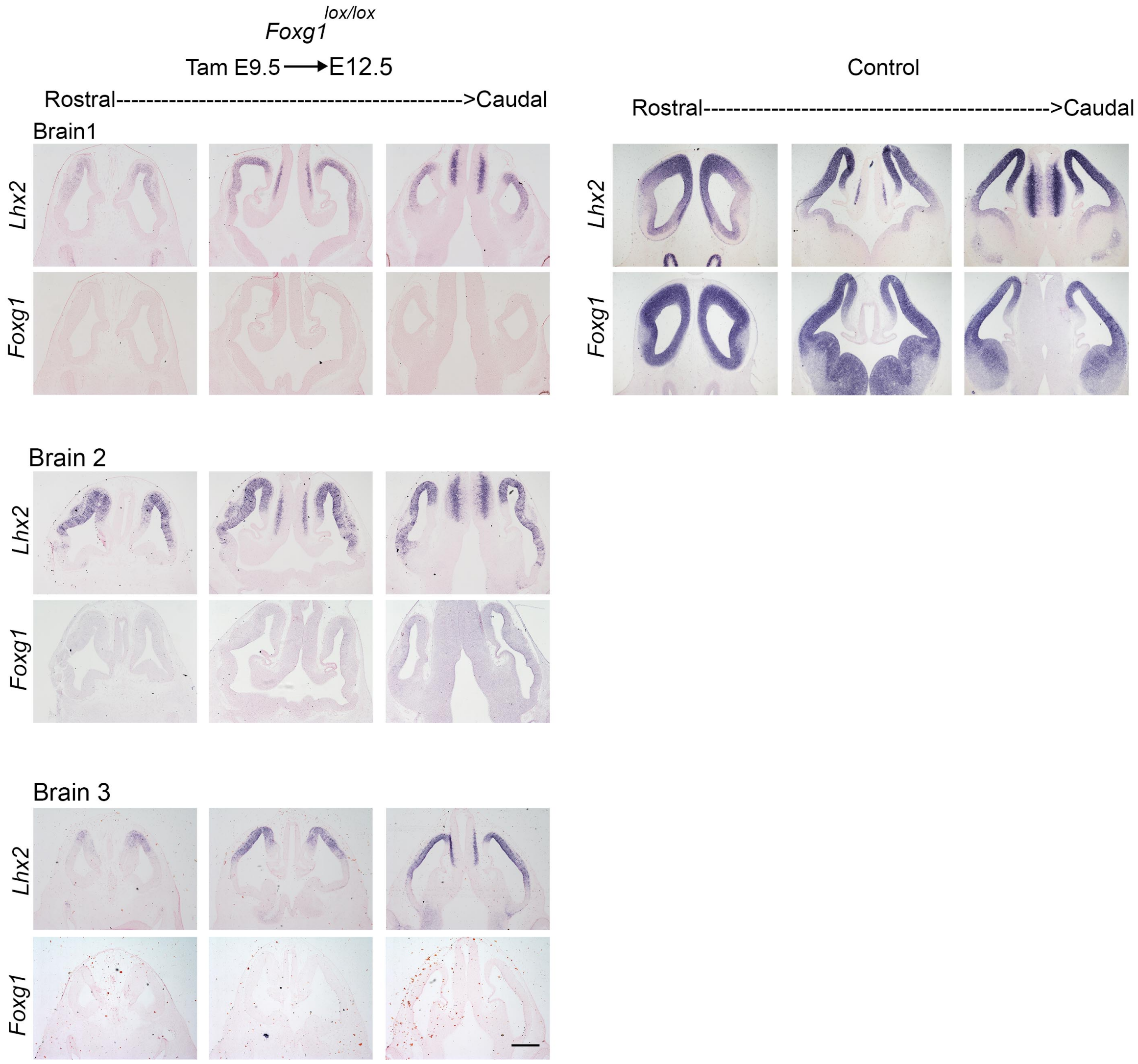

Figure S4: *Lhx2* expression is reduced in the absence of *Foxg1* (Additional embryos corresponding to Figure 2 E,F)

Tamoxifen (Tam) was administered at E9.5 to control and *CreERT2; Foxg1*$^{lox/lox}$ embryos and brains were harvested at E12.5. Panels shows sections from three embryo brains displaying the rostro-caudal expression profiles of *Lhx2* in *Foxg1* conditional mutants; In serial sections, a probe against *Foxg1* reveals near-complete recombination since the expression is undetectable. Scale bar is 200µm.

Figure S5

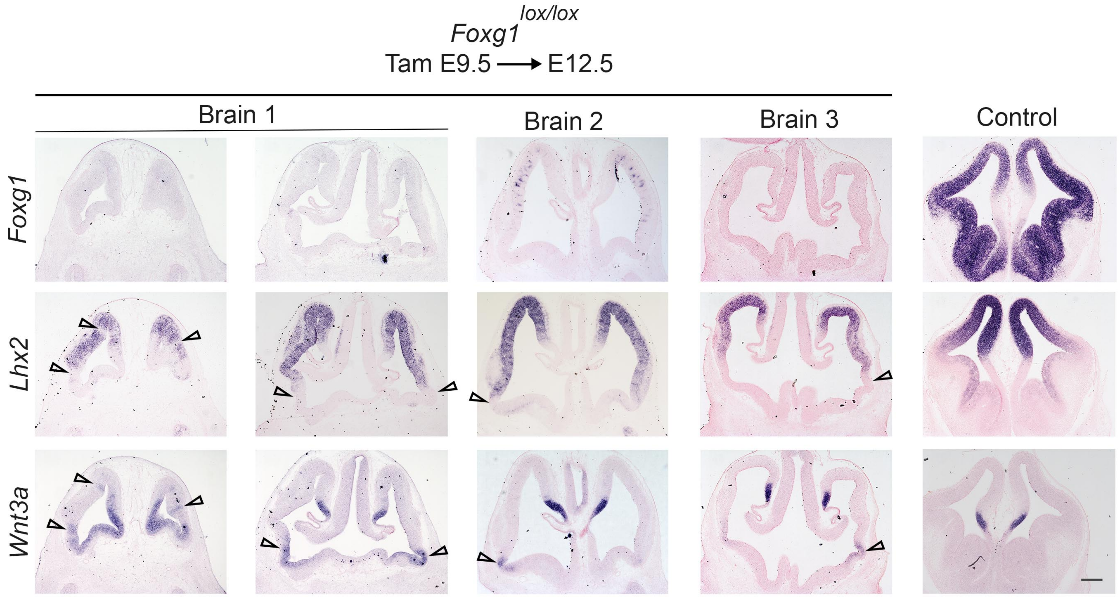

Figure S5: Additional embryos corresponding to Figure 3 (D-F) in which tamoxifen was administered to *CreERT2; Foxg1*$^{lox/lox}$ embryos at E9.5 and brains were harvested at E12.5. Scale bar is 200µm.

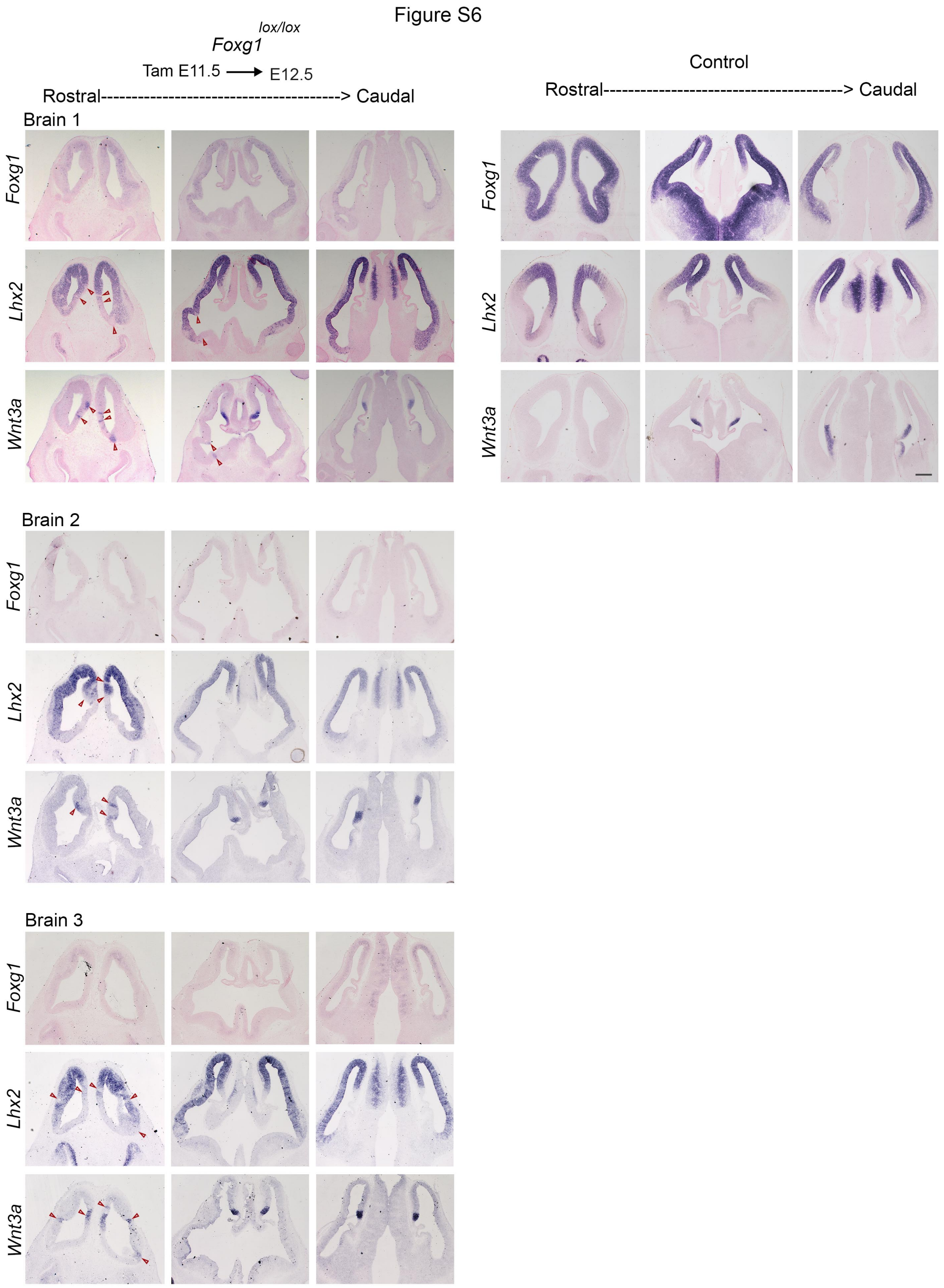

Figure S6: Additional embryos corresponding to Figure 3 (G-I) in which tamoxifen was administered to *CreERT2; Foxg1$^{lox/lox}$* embryos at E11.5 and brains were harvested at E12.5. Scale bar is 200μm.

Figure S7

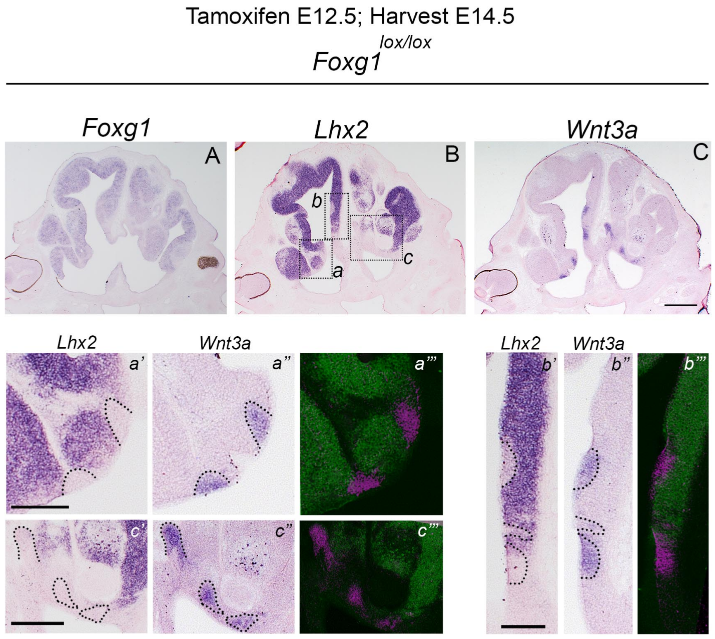

Figure S7: An additional embryo corresponding to Figure 3 (J-L) in which tamoxifen was administered to *CreERT2; Foxg1*$^{lox/lox}$ embryos at E12.5 and brains were harvested at E14.5. (A-C) shows the expression of *Foxg1* (A), *Lhx2* (B), and hem marker *Wnt3a* (C), in *Foxg1* conditional mutant brains. Corresponding regions of B and C marked by rectangles a and b are compared using false-color overlays (a'-a''' and b'-b''') demonstrating that *Wnt3a* appears in *Lhx2*-negative patches (identified by dotted lines). Scale bar is 400µm for A-C; 200um for a'-a''', c'-c''' and 100µm for b'-b'''.

Development • Supplementary information

Figure S8

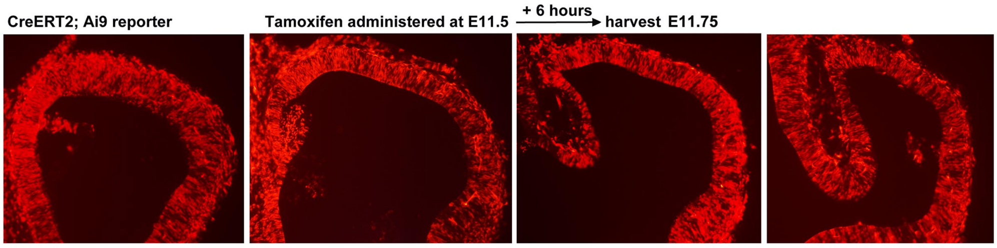

Figure S8: Timing of tamoxifen action in the dorsal telencephalon:

Tamoxifen was administered to pregnant dams carrying the Ai9 reporter allele, at E11.5, and the embryos were harvested 6 hours later (E11.75). The Ai9 reporter is robustly expressed all over the telencephalon, indicating that Cre action is well underway 6 hours post tamoxifen administration in this tissue.

Supplementary Table 1

Loss of **Lhx2** From (stage)	Hem	Cortical primordium medial / lateral	Antihem	Ventral telencephalon	Reference	Schematic 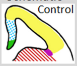 Control
E8.5 or earlier	expanded; *Foxg1* not expressed	not present	expands; *Foxg1* expressed	present; *Foxg1* expressed	Mangale et al, 2008; this study	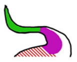
E9.5	expanded marginally; *Foxg1* not expressed	present medially; *Foxg1* expressed	expands; *Foxg1* expressed	present; *Foxg1* expressed	this study	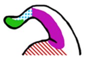
E10.5 or later	no expansion	present medially and laterally; *Foxg1* expressed	no expansion	present; *Foxg1* expressed	this study	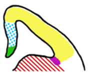

Loss of **Foxg1** From (stage)	Hem	Cortical primordium medial / lateral	Antihem	Ventral telencephalon	References	Schematic 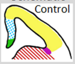 Control
E8.5 or earlier	expanded and seen in ectopic patches; *Lhx2* lost in the same patches	only medial cortical primordium present	not present	specified but undergoes cell death and is not present by E12.5	Muzio and Mallamaci, 2005	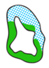
E9.5	expanded (or seen in ectopic patches); *Lhx2* lost in the same patches	only medial cortical primordium present	not present	specified but undergoes cell death and is not present by E12.5	this study, Xuan et al 1995, Huh et al 1999	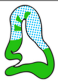
E10.5 or later	expanded (or seen in ectopic patches); *Lhx2* lost in the same patches	medial and lateral cortical primordium present	not present	some ventral tissue appears to be present at E12.5	this study	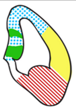
